# Factors affecting DH plants in vitro production from microspores of European radish

**DOI:** 10.18699/VJ20.592

**Published:** 2020-02

**Authors:** E.V. Kozar, E.A. Domblides, A.V. Soldatenko

**Affiliations:** Federal State Budgetary Scientific Institution “Federal Scientific Vegetable Center”, VNIISSOK, Odintsovo region, Moscow oblast, Russia; Federal State Budgetary Scientific Institution “Federal Scientific Vegetable Center”, VNIISSOK, Odintsovo region, Moscow oblast, Russia; Federal State Budgetary Scientific Institution “Federal Scientific Vegetable Center”, VNIISSOK, Odintsovo region, Moscow oblast, Russia

**Keywords:** DH plants, Raphanus sativus, in vitro microspore culture, embryogenesis factors, regeneration from in vitro culture, heat treatment, androgenesis, DH-растения, Raphanus sativus, культура микроспор in vitro, факторы эмбриогенеза, регенерация в культуре in vitro, температурная обработка, андрогенез

## Abstract

Over the recent years the market demand for scaling up the production of European radish (Raphanus sativus L.) varieties and hybrids for open and protected production, varying in ripeness group, root shape and color, has drastically increased. Therefore, the expansion of genetic diversity and acceleration of the selection process are important. Doubled haploid technology considerably curtails the time required for creation of homozygous constant parental cell lines when in vitro microspore culture is used as the most promising method. For the first time, we were able to realize the full production cycle of DH plants of European radish by in vitro microspore culture up to inclusion of the produced material into the selection process. We have selected: preferable flower bud size, heat shock parameters, induction and regeneration media. It was revealed that linear length on the flower buds with the best possible stage of microspore development is genotype-specific: the flower bud length 2.8–3.3 mm is optimal for accessions of Rhodes and 3.7–4.2 mm is optimal for accessions of Teplichny Gribovsky. Heat shock at 32 °C for 48 hours is the most suitable for most genotypes. For the first time Murashige and Skoog based culture medium has been used for embryogenesis induction, and a major dependence of embryogenesis induction on the genotype × medium interaction was found. At regeneration and tiller stage it is advisable to add 1 mg/mL of benzylaminopurine and 0.1 mg/L of gibberellic acid to the medium, and rotting of micro-sprouts is performed with the use of hormone-free medium. Analysis of the produced regenerant plants by chromosome count and cell nucleus flow cytometry showed that 69 % of plants have a diploid chromosome set, 9 % have a haploid chromosome set, and 22 % have mixoploids and aneuploids chromosome sets. The seed progeny from doubled haploids and mixoploids were obtained by self-pollination, where all R1 plants had a doubled set of chromosomes. This study launches the development of an efficient method of radish doubled haploid production to be used in the selection process.

## Abstract (ru)

В последние годы резко повысилась потребность рынка в увеличении производства сортов и гибридов редиса европейского (Raphanus sativus L.) для открытого и закрытого грунта, разнообразных по группам спелости, форме и окраске корнеплода. Поэтому важно расширять генетическое разнообразие и ускорять селекционный процесс. Технология получения удвоенных гаплоидов существенно сокращает время при создании гомозиготных константных родительских линий, для получения которых наиболее перспективен метод культуры микроспор in vitro. Нам впервые удалось осуществить полный цикл получения DH-растений редиса европейского в культуре микроспор in vitro, до включения материала в селекционный процесс. Подобраны: оптимальный размер бутонов, параметры теплового шока, среды для индукции и регенерации. Выявлено, что линейная длина бутонов с оптимальной стадией развития микроспор генотип-специфична. Так, для сортообразца Родос оптимальным является показатель 2.8–3.3 мм, а для сортообразца Тепличный Грибовский – 3.7–4.2 мм. Для большинства генотипов оптимален температурный шок 32 °C в течение 48 ч. Впервые для индукции эмбриогенеза использована модифицированная среда Mурасиге–Скуга и обнаружено существенное влияние взаимодействия факторов «генотип × среда» на индукцию эмбриогенеза. Для этапа регенерации растений из эмбриоидов рекомендуется добавление к среде 1 мг/л бензиламинопурина и 0.1 мг/л гиббереллиновой кислоты, укоренение микропобегов проводится на безгормональной среде. Анализ полученных растений-регенерантов методом подсчета хромосом и методом проточной цитометрии клеточных ядер показал, что 69 % растений имели диплоидный набор хромосом, 9 % – гаплоидный, 22 % – миксоплоидный и анеуплоидный. Семенное потомство удалось получить самоопылением из удвоенных гаплоидов и миксоплоидов, причем все растения R1 имели удвоенный набор хромосом. Это исследование является начальным этапом в разработке эффективной методики получения удвоенных гаплоидов редиса для применения в селекционном процессе.

## Introduction

In modern crop breeding, the priority is to create F1 hybrids that differ from the cultivars in high yield and evenness of plants in terms of ripening and quality of productive organs. The most difficult, time-consuming, and lasting link in this process is to derive constant parental lines that take 6 to 12 years to create using traditional selection methods. In most developed countries, technologies for double haploid production (DH technologies) are currently widely used to accelerate breeding (Dunwell, 2010), which makes it possible to accelerate the selection process by at least 3–4 years (Ferrie, Möllers, 2011).

The main methods for obtaining haploids and their classification are considered in a number of reviews (Maluszynski et al., 2003; Dunwell, 2010; Asif, 2013). Haploid technologies expand the spectrum of the morphogenetic process, facilitate the selection of useful genes, facilitate the detection of rare recessive alleles, help create unique forms, and thus increase the efficiency of practical selection (Forster, Thomas, 2005). In the largest foreign breeding campaigns (Syngenta, Bayer, etc.), obtaining doubled haploids of some plant species is already a routine and necessary stage of selection. In Russia, successes in this area have also been achieved in a number of grains (Ignatova, 2011) and vegetables (Pivovarov et al., 2017; Vjurtts et al., 2017). By the centenary year in 2020 from the establishment of Federal Scientific Vegetable Centre (VNIISSOK) hybrids in major vegetable crops such as head cabbage, broccoli, sweet pepper, winter squash and others have been developed with the use of doubled haploids (Domblides al., 2017).

Doubled haploids can be obtained on the basis of androgenesis (anther culture or microspore culture), gynogenesis (culture of unfertilized ovules) and parthenogenesis (pollination of irradiated/chemically treated pollen or pollen of distant species). The success of these technologies is determined by two processes: the induction of embryogenesis from microspores/haploid cells of the embryo sac and the regeneration of plants from embryoids. These processes are influenced by a large number of factors: conditions for growing donor plants, genotype, stage of development of microspores/cells of the embryo sac, pretreatment of buds and microspores, culture media and cultivation conditions (Ferrie, Caswell, 2011). In view of this, it is impossible to develop a universal method for producing DH plants and it is necessary to optimize individually for each species, and even cultivars. Cellular technologies are actively developing; however, the literature describes a very limited number of effective protocols for obtaining doubled haploids of vegetable cultures of the family Brassicaceae Burnett, some of which are protected by patents. The main problem is the low yield of DH plants, therefore, increasing the effectiveness of the methods is very significant and attention is paid to this issue around the world.

In vitro microspore culture (androgenesis) takes a leading place in breeding programs to accelerate the creation of highly productive hybrids and varieties of agricultural plants. Under certain conditions, isolated microspores (the optimal combination of culture conditions and stress exposure) can be transferred from the normal gametophytic to sporophytic pathways, resulting in the formation of embryoids that transform into haploids (Hs), doubled haploids (DH plants), mixoploids and aneuploids. The absence of microspores of somatic tissues in the culture allows us not to question the origin of the obtained plants (Domblides et al., 2016).

The first successful microspore culture studies in the Brassicaceae family were conducted in the early 1980s (Lichter, 1982). Later, a basic protocol of microspore culture for rape was developed, which serves as the basis for DH technology for plants of the genus Brassica L. (Pechan, Keller, 1988). Then, microspore culture began to be used for various varieties of cabbage: cauliflower (B. oleracea var. botrytis), broccoli (B. oleracea var. italica), semi-loose and loose cabbage (B. oleracea var. сostata), kohlrabi (B. oleracea var. gongylodes), ornamental cabbage (B. oleracea var. acephala) and white cabbage (B. oleracea var. capitata), as well as Chinese cabbage (B. rapa ssp. chinensis) (Lichter, 1989; Cao et al., 1990; Takahata, Keller, 1991; Duijs et al., 1992; Zhang et al., 2008; Winarto, Teixeira da Silva, 2011; Yuan et al., 2012). Published protocols for the family Brassicaceae are given in the review (Maluszynski et al., 2003). Unfortunately, the experimental approaches described in the literature cannot always be reproduced, and the lack of standard methods often contributes to the appearance of conflicting results.

European radish (Raphanus sativus L.) is a root plant of the cabbage family. This is one of the most precocious and economically significant vegetable crops. However, there is currently no effective technique for producing doubled haploids for radishes. There is only a small number of publications (Takahata et al., 1996; Chun et al., 2011; Han et al., 2014, 2018; Tuncer, 2017) on the application of the in vitro method of microspore culture for radishes. Still, none of the studies completed the full cycle of obtaining DH plants in the culture of European radish microspores. Now there is no clear idea of the main reasons for the difficulty in obtaining doubled radish haploids, and at the moment they can only be identified empirically.

The aim of our research is to develop a technology for producing DH plants of European radish to include the obtained linear material in the breeding process with the study and a detailed description of the problems at each stage.

## Materials and research methods

**Research material and conditions for growing donor plants.** In our work we used varieties of European radish from the laboratory collection of table root crops of the Federal State Budgetary Scientific Institution “Federal Scientific Vegetable Center”, Moscow Region:

**Table:**
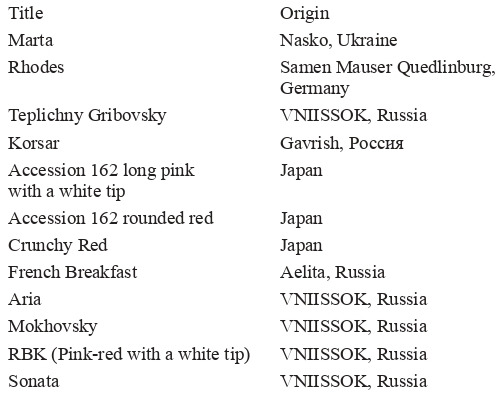
3

Donor plants were grown in a vegetation chamber with light bulbs (Osram plantstar 600 W) at a constant temperature of 19 °C, illumination of 9000 lux at a 16-hour photoperiod, to stimulate flowering.

**Study of the stages of development of microspores.** The cytological studies were performed to study the relationship between the size of the bud and the stage of development of microspores. A differential staining technique (Alexander, 1969) and an Axio Imager A2 microscope (Carl Zeiss, Germany) were used for visualization of pollen and microspores.

**Induction of embryogenesis in microspore culture.** A tech-nique developed in the biotechnology laboratory (FSBSI “Federal Scientific Vegetable Center”) for the culture of microspores of the family Brassicaceae (Domblides et al., 2016) with various media options: NLN-13 (Lichter, 1982) and MS (Murashige, Skoog, 1962) with 13 % sucrose and 500 mg/L casein hydrolyzate – for the induction of embryogenesis – was taken as a basis. SIGMA reagents marked “plant cell culture tested” were used for the experiments.

**The temperature treatment** took place immediately after the introduction of microspores to the in vitro culture in a thermostat at 32 °C for one to four days. This study used the optimal medium for the induction of embryogenesis, as defined in a previous experiment on the induction of embryogenesis in microspore culture. The study of the optimal heat treatment with a varying duration of temperature stress was carried out five times for each individual sample.

**Obtainment of regenerated plants.** Embryoids at the stages of large globules, as well as heart-shaped and torpedoshaped, were placed in Petri dishes on the hormone-free medium Murashige–Skoog (MS). For germination, the embryoids were transferred onto the following media: (1) MS with 2 % sucrose, 0.1 mg/L benzylaminopurine (BAP) and 3.0 g/L phytogel (Sigma, USA); (2) MS with 2 % sucrose, 1 mg/L BAP, 0.1 mg/L gibberellic acid (GA), 3.0 g/L phytogel; (3) MS with 2 % sucrose, 0.2 mg/L thidiazuron (N-phenyl-N′- (1,2,3-thiadiazole-5yl)urea) (TDZ). The resulting sprouts and embryoids were separated and transferred to a hormone-free MS medium with 2 % sucrose and 3.0 g/L phytogel, pH = 5.8 for rooting. Cultivation was carried out on racks with fluorescent lamps, with a photoperiod of 14 hours, illumination of 2500 lux, at a constant temperature of 25 °C.

**The growing of regenerated plants.** Plants with normally developed leaves and root system were transferred to vegetation vessels filled with a mixture of peat and perlite (7:3), covered with perforated plastic cups to adapt plants to in vivo conditions. Regenerant plants were grown under the same climacteric conditions as donor plants.

**The ploidy of regenerated plants** was determined by flow cytometry of cell nuclei. It was performed on the basis of the bioengineering laboratory of Altai State University in Barnaul using a Partec CyFlow PA flow cytometer (Partec GmbH, Germany) with a laser radiation source and a wavelength of 532 nm.

**Statistical analysis** was performed using ANOVA: One way ANOVA, Factorial ANOVA, and Fisher Test.

## Results and discussion

**Determination of the dependence of the stage of development of microspores and the yield of embryoids on the size of the buds**

Studies have shown that the microspore population structure in the anthers of European radish buds is very heterogeneous and is represented by microspore fractions at different stages of development in one bud. This is consistent with data from other authors (Takahata et al., 1996; Bhatia et al., 2018).

It is believed that microspores are most susceptible to external factors and are able to change the development path at the late vacuolated unicellular and early bicellular stages (Pechan, Keller, 1988). Therefore, it is important to maximize the concentration of these stages in the culture, for which it is necessary to determine the linear size of the buds that contains such stages of development of microspores and pollen in maximum concentration. Table 1 presents the results of the analysis of the percentage (fraction) of microspores at the susceptible stage of development in buds of different sizes (from 2.5 to 6.0 mm) in four radish genotypes that showed responsiveness to embryogenesis.

**Table 1.**
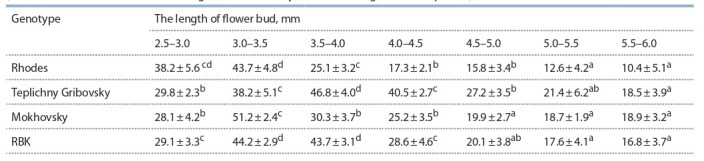
Relationship between the length of European radish flower bud and the sensitive microspore level (at the late one-celled vacuolated stage and at the early two-celled stage of development) Note: Values with the same letters do not differ significantly at p < 0.05.

It was revealed that the percentage of susceptible microspores in radish buds rarely reaches 50 %. For comparison, in buds of responsive cultures, the proportion of such microspores reaches 80 %, for example in B. oleracea var. capitata L. (Bhatia et al., 2018). It was noted that the optimal bud sizes for different radish genotypes significantly differ in terms of the responsiveness of microspores to the induction of embryogenesis in an in vitro culture.

**Fig. 1.**
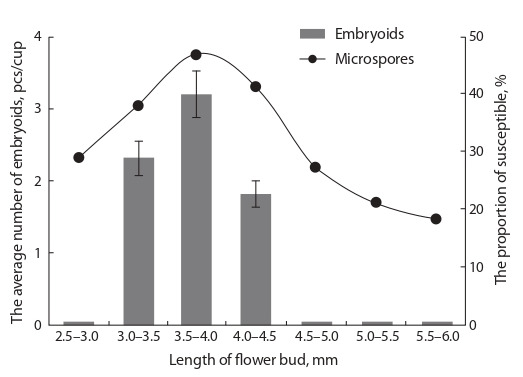
Dependence of embryoid production on the flower bud size and microspore percentage at the susceptible stage of development of the European radish cv. Teplichny Gribovsky (average and variations by replicates).

To identify the significance of the qualitative composition of isolated microspores for the induction of embryogenesis, we evaluated the yield of embryoids in an in vitro microspore culture, isolating microspores from buds of various sizes using the example of the Teplichny Gribovsky variety (Fig. 1). The experiment was repeated five times: we selected buds ranging in size from 2.5 to 6 mm in 0.5 mm increments and then incubated microspores on standard NLN-13 medium (Lichter, 1982) with 13 % sucrose, pH 5.8.

Thus, as a result of the experiment, it was confirmed that when the bud size contains the highest percentage of microspores at the susceptible stage (on average 44–51 % depending on the genotype), the yield of embryoids is maximum. With a change in the percentage of susceptible microspores by 6–8 %, a sharp decrease in the yield of embryoids is observed, and when the percentage of susceptible microspores falls below 30 %, regardless of the size of the buds, the formation of embryoids does not occur. Similar results were obtained for other varieties of radish.

As a result, the most optimal bud size for inducing embryogenesis in an in vitro culture was selected for each studied European radish genotype.

**Influence of the composition of the nutrient medium and the duration of heat treatment on the induction of embryogenesis in a culture of radish microspores**

The transition to the sporophytic path of development does not occur spontaneously, for this it is necessary to achieve the creation of optimal conditions, among which the greatest influence is: heat treatment, cultivation conditions and the composition of nutrient media for the induction of embryogenesis. The selection of nutrient media for the experiments was based on published data, so NLN-13 media (Lichter, 1982; Chun et al., 2011; Han et al., 2014) or 1/2 norms of macronutrients NLN-13 are usually used to induce embryogenesis of plants of the family Brassicaceae (Takahata et al., 1996; Tuncer, 2017; Han et al., 2018), which include the amino acids glutamine and serine, which have a positive effect on embryogenesis. It is also known that a significant role in stimulating somatic embryogenesis in suspension culture is played by casein hydrolysate, which is a mixture of various amino acids and is used in nutrient media for table carrots (Masuda et al., 1981; Vjurtts et al., 2017). In view of this, we decided to use MS medium for the first time with the addition of casein hydrolysate in an in vitro microspore culture for radish.

In our experiments, none of the studied genotypes formed embryoids using the medium 1/2 norms of macronutrients NLN-13, and therefore, in subsequent experiments, this medium was not used. When incubating radish microspores on standard NLN-13 and MS medium with casein hydrolysate, the embryogenesis process was induced in four out of twelve varieties (Table 2).

**Table 2.**
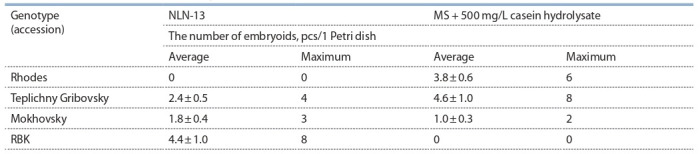
Effect of the culture medium composition on embryogenesis (yield of embryoids) in microspore culture of different genotype of the European radish Note: Significant difference: Factor А (variety): Fobserved 4.2 > Ftheor. 2.9; Factor В: Fobserved 0.2 < Ftheor. 4.1; А × В interaction: Fobserved 17.1 > Ftheor. 2.9.

**Fig. 2.**
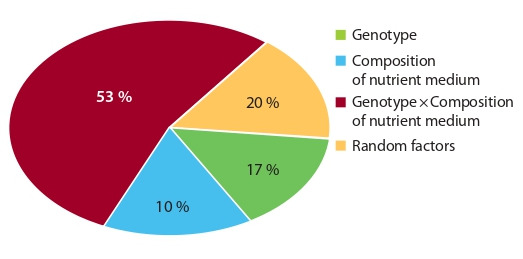
Contribution of the factors “genotype”, “composition of nutrient medium”, and the interaction of respective factors on in vitro embryogenesis induction of European radish in microspore culture.

The use of NLN-13 and MS media with casein hydrolysate showed a distinct genotype-specific responsiveness of embryogenesis induction in radish culture to the medium composition, which was mainly determined by the interaction of the factors “genotype” and “environment” with a share of influence of more than 50 % in this experiment (Fig. 2).

It was not possible to get embryoids from the Rhodes variety on standard NLN-13 medium, but on MS medium with a casein hydrolysate, their yield was up to six embryoids per Petri dish; for Teplichny Gribovsky, it was also more suitable, for the maximum yield of embryoids was eight embryoids on a Petri dish. For the Mokhovsky and RBK variety samples, the best results were obtained on standard NLN-13 medium, and the maximum yield reached three and eight embryoids per Petri dish, respectively.

**Fig. 3.**
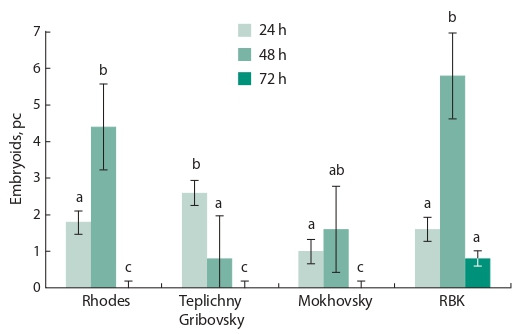
Effect of heat treatment time of the isolated microspores in an incubator at 32 °С on embryoid production (embryoid/Petri dish) in microspore culture by the European radish genotypes. Significant difference: Factor А (variety): Fobserved 11.1 > Ftheor. 2.7; Factor В (heat treatment time): Fobserved 58.9 > Ftheor. 2.8; А × В interaction: Fobserved 9.8 > Ftheor. 2.0.

In order to initiate the process of switching microspores from the gametophytic pathway to the sporophytic pathway, they are stressed by high temperature. Isolated microspores either stop in their development and die, or continue to develop along the gametophyte pathway. Temperature stress is applied at the stage preceding the first haploid mitosis or during it, which usually occurs during the first eight hours after the introduction of microspores to the culture, therefore they are critical. Choosing the optimal regime for each individual sample, we analyzed the effect on embryogenesis by the temperature treatment of isolated microspores in a thermostat at 32 °C for one to four days immediately after the start of cultivation (Fig. 3).

So, for ‘Rhodes’, ‘Mokhovsky’ and ‘RBK’, the processing was optimal for two days, and only for one day for ‘Teplichny Gribovsky’. Incubation of embryoids for three days led to a slowdown in the rate of development of embryoids in the RBK variety specimen, and their complete absence in other variety specimens. When the time of heat treatment was increased to 4 days, the formation of embryoids did not occur in any genotype.

**Fig. 4.**
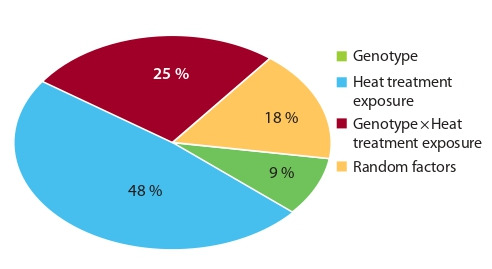
Contribution of the factors “genotype”, “heat treatment exposure”, and the interaction of respective factors on in vitro embryogenesis induction of European radish in microspore culture.

Within each genotype, the proportion of the effect of the duration of the heat treatment (factor B) on the yield of embryoids was highly significant and amounted to 48 %, and varietal specificity was noted due to the interaction of the factors (DW 25 %) “genotype” and “duration of heat treatment” (Fig. 4).

**Fig. 5.**
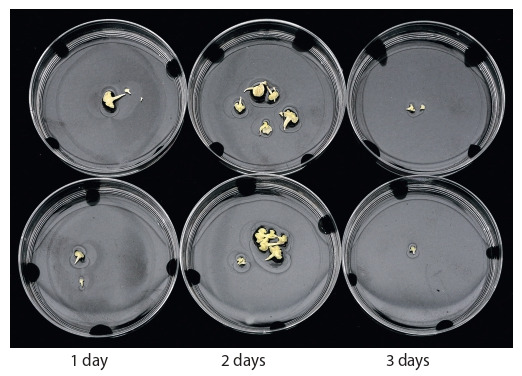
Effect of heat treatment time of the isolated microspores in an incubator at 32 °С on embryogenesis (yield of embryoids) in microspore culture of European radish variety ‘RBK’ on NLN-13 culture.

Figure 5 shows, depending on the duration of temperature stress, the most obvious results of the formation of embryoids within one variety sample in a microspore culture in vitro, using the example of the European radish variety ‘Pink-red with a white tip’ (RBK).

In this variety after treatment of temperature within one day, the maximum yield was reached three embryoids per Petri dish, after treatment for two days yield was up to eight well-developed embryoids, and within three days – up to two underdeveloped embryoids per Petri dish.

**Development of a scheme for the regeneration of embryoids obtained in microspore culture in vitro**

Embryoids were transferred to solid nutrient media for regeneration after the embryoid culture step in liquid media to induce embryogenesis. There was no direct germination of embryoids into regenerant plants, therefore, at the initial stage, it is necessary to start the process of secondary embryogenesis and the formation of secondary growth points with subsequent shoot formation (Shumilina et al., 2015; Domblides et al., 2016). Sometimes secondary embryoids and growth points were formed on a hormone-free medium, but various plant growth regulators are used for additional stimulation. The following solid nutrient media were used in our experiment: MS is hormone-free, MS with 1 mg/L BAP; MS with 0.2 mg/L TDZ; MS with 0.1 mg/L GA and 1 mg/L BAP.

**Fig. 6.**
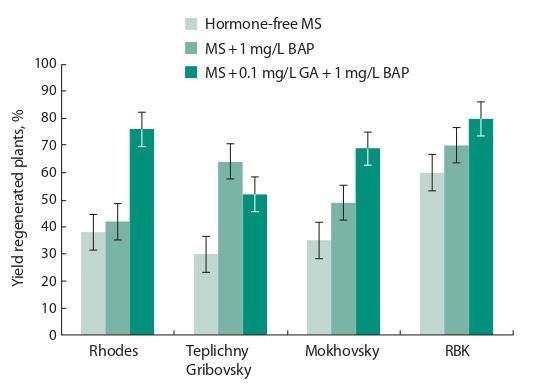
European radish regenerant plant survival rate at the first regeneration stage depending on culture medium composition and the genotype.

The frequency of formation of secondary growth points and embryogenesis with subsequent shoot formation was ranging from 30 to 80 %, depending on the genotype and composition of the nutrient medium. In the majority of variety specimens, the best results were obtained on media with the combined addition of BAP and GA (from 69 to 80 %). An exception was the ‘Teplichny Gribovsky’ sample, where the best result (up to 63 %) was obtained on a medium with the addition of BAP (Fig. 6).

The inclusion of tiadizuron in the medium gave a negative effect, although there are articles describing successful experiments on regeneration on MS medium: 0.8 % agar, 3 % sucrose, 0.2 mg/L TDZ (Bunin, Shmykova, 2004). In our experience, all embryoids transplanted onto this medium darkened and stopped their development within three to five days.

Due to the lack of direct embryogenesis, a separate rooting stage is required for the formed shoots and buds. For this, the formed buds and shoots were transferred onto a solid hormone-free MS medium. The formation of a normally developed root system was rare. In the majority of embryoids, the lower part of the epicotyl began to thicken, forming callus structures with poorly developed roots; such plants did not take root well in vivo.

An analysis of the obtained regenerant plants by flow cytometry of cell nuclei showed that 69 % of the plants were doubled haploids, 9 % were haploids and 22 % were mixoploids and aneuploids. In doubled haploids and some mixoploids, seedlings were obtained by self-pollination, in which all R1 plants had a diploid set of chromosomes (2n = 2c = 18).

## Conclusion

A change in the development path of microspores depends on many factors, the degree of influence of each of which for different cultures can differ significantly.

One of the most important factors for European radish is the stage of development of microspores in buds. It has been shown that for the European radish the linear size of buds that contain the maximum concentration of microspores at the optimal stage of development for embryogenesis is genotype-specific. In view of this, it is necessary to carry out reconnaissance determination of the optimal bud size by studying the qualitative composition of microspores in buds of various lengths for each individual genotype.

Also, a distinct genotype-specific responsiveness to embryogenesis was established in the culture of European radish, and the influence of medium composition on the intensity of embryogenesis with a high degree of interaction of these factors was found. Therefore, different induction media should be tested for each variety sample. Thus, embryogenesis was induced in the ‘RBK’ specimen only on standard NLN-13 medium (Lichter, 1982) supplemented with activated carbon recommended for species of the genus Brassica L. (Domblides et al., 2016), and in the ‘Rhodes’ specimen on MS medium with 13 % sucrose and 500 mg/L casein hydrolysate used for root crops of the genus Daucus L. In other cultivars, embryogenesis was observed on both media, but with a different yield of embryoids: in ‘Teplichny Gribovsky’, the yield on MS medium with casein hydrolysate was higher and amounted to 8 embryoids per Petri dish (160 embryoids/ 100 buds); in ‘Mokhovsky’, the highest yield was up to 3 embryoids per Petri dish (60 embryoids/100 buds) on NLN-13 medium.

The effect of heat shock duration at 32 °C on the induction of embryogenesis is also genotype-specific. However, when using an optimally selected medium for each variety sample, the influence of the duration of heat treatment showed a general trend. For most genotypes, temperature shock within 48 hours is optimal. Incubation of microspores for more than two days leads to a slowdown in the rate of development of embryoids, and an increase in the duration of temperature treatment to four days leads to a complete inhibition of the process, i. e., the search for the optimum duration of heat shock for different radish genotypes can be narrowed to one or two days.

Despite the optimization of individual elements of the technology (bud size, medium composition and duration of heat treatment), the embryoid yield of responsive European radish genotypes was quite low and did not exceed 160 pieces per 100 buds. One of the reasons is the uneven development of microspores in buds, which initially limits the potential for multiple formation of embryoids, due to the low proportion of microspores capable of embryogenesis (≤ 51.2 %). In particular, microspores that do not switch to the sporophytic pathway of development die, which entails the formation of toxins and a change in the pH of the medium (Chun et al., 2011; Shmykova et al., 2015), hindering the normal development of embryoids. The literature contains data on the addition of various toxins to the induction media (Chun et al., 2011; Shmykova et al., 2015) and buffer compounds to stabilize the pH level. But when using various additives, there is a problem of adsorption from the environment along with toxins of the necessary substances for embryogenesis. Therefore, it is necessary to find a way to effectively separate the microspore population into fractions according to the stages of development even before their introduction into in vitro culture.

At the stages of regeneration and rooting of DH plants for European radish, it was revealed that it is possible to start and stabilize the processes of secondary embryogenesis using growth regulators. But it is important to consider that the direction and effectiveness of some of them may differ significantly from the results previously given in the literature (Bunin, Shmykova, 2004). So, in our experiments, the best positive results were obtained by the simultaneous introduction of BAP and GA growth regulators into the MS, and the addition of TDZ led to a negative effect.

At the stage of root formation and rooting, the greatest number of regenerated plants perished. Due to the biological characteristics of development, radish is very sensitive to damage of the growth point of the main root. That is, in addition to the composition of the nutrient medium, it is necessary to improve elements of the technique for transferring embryoids to a solid nutrient medium and methods for planting regenerated plants in the soil. Also, the advantage of obtaining plants through secondary embryogenesis should be noted: several radish plants can be obtained from each embryoid, and in this case spontaneous doubling of chromosomes occurs more often, which makes it possible to carry out a more complete assessment in the first generation and collect more seeds.

The resulting regenerant plants in an in vitro microspore culture were diverse in terms of ploidy level. Most of the plants were doubled haploids, one fifth of them were mixoploids and aneuploids, and some of the mixoploids were fertile and set seeds. Moreover, all plants (R1) obtained by self-pollination of mixoploids went into a diploid form. In view of this, in the culture of the European radish, mixoploid plants can also be valuable and be suitable for inclusion in the breeding process. All obtained seed offspring is included in the breeding process under the guidance of the laboratory of root crops (FSBSI “Federal Scientific Vegetable Center”).

**Fig. 7.**
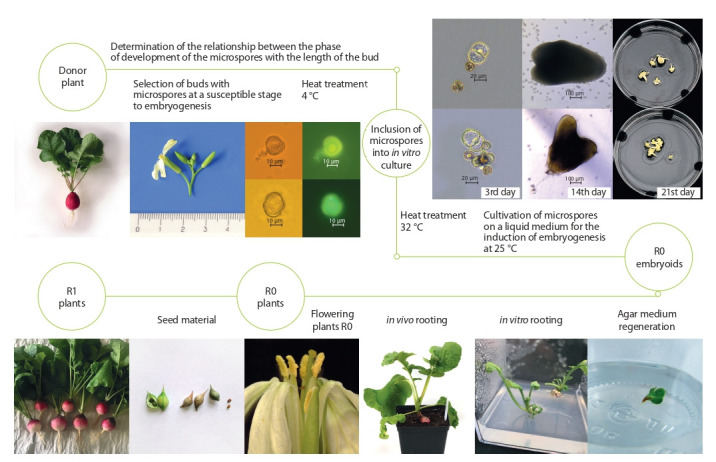
Full cycle of obtaining doubled haploids in an in vitro microspore culture for European radish, using the example of RBK strainer (Pink-red with a white tip).

Thus, for the first time, we were able to complete the full cycle of obtaining doubled radish haploids in an in vitro microspore culture (Fig. 7), we were able to identify the main problems in obtaining doubled haploids and outline the direction for further research to develop methods for creating high-efficiency DH plants.

## Conflict of interest

The authors declare no conflict of interest.
